# Molecular mechanisms of inhibitor of growth (ING) family members in health and malignancy

**DOI:** 10.1186/s12935-022-02693-w

**Published:** 2022-09-02

**Authors:** Mohammad Taheri, Bashdar Mahmud Hussen, Sajad Najafi, Atefe Abak, Soudeh Ghafouri-Fard, Majid Samsami, Aria Baniahmad

**Affiliations:** 1 Institute of Human Genetics, Jena University Hospital, Jena, Germany; 2 Urology and Nephrology Research Center, Shahid Beheshti University of Medical Sciences, Tehran, Iran; 3 Department of Pharmacognosy, College of Pharmacy, Hawler Medical University, Kurdistan Region, Erbil, Iraq; 4 Center of Research and Strategic Studies, Lebanese French University, Erbil, Kurdistan Region Iraq; 5 Department of Medical Biotechnology, School of Advanced Technologies in Medicine, Shahid Beheshti University of Medical Sciences, Tehran, Iran; 6 Skull Base Research Center, Loghman Hakim Hospital, Shahid Beheshti University of Medical Sciences, Tehran, Iran; 7 Department of Medical Genetics, School of Medicine, Shahid Beheshti University of Medical Sciences, Tehran, Iran; 8 Cancer Research Center, Shahid Beheshti University of Medical Sciences, Tehran, Iran

**Keywords:** ING, Tumor suppressor gene, Cancer, Oncogene, Cellular senescence, Splice variants

## Abstract

*ING* genes belong to family of tumor suppressor genes with regulatory functions on cell proliferation, apoptosis, and cellular senescence. These include a family of proteins with 5 members (ING1-5), which are downregulated in human malignancies and/or affected by pathogenic mutations. ING proteins are highly evolutionarily conserved proteins containing several domains through which bind to chromatin structures by exerting their effects as readers of histone modification marks, and also binding to proteins like p53 involved in biological processes such as cell cycle regulation. Further, they are known as subunits of histone acetylation as well as deacetylation complexes and so exert their regulatory roles through epigenetic mechanisms. Playing role in restriction of proliferative but also invasive potentials of normal cells, INGs are particularly involved in cancer development and progression. However, additional studies and experimental confirmation are required for these models. This paper highlights the potential impact that INGs may have on the development of human cancer and explores what new information has recently arise on the functions of ING genes.

## Introduction

The family of tumor suppressors known as the Inhibitor of Growth (ING) proteins has five conserved genes in humans and mice, with the majority of these genes generating several proteins via alternative splicing [1]. The key factors encouraging cancer formation and progression are the inactivation of some genes and the activation of others [2, 3]. Notably, 3 categories of gene sets playing role in critical cellular functions are known to be affected by accumulating mutations and involved in human malignancies.

Accumulating evidence shows that pathogenic mutations damage these groups and turn them into sequences with changed/inactivated functionality. Firstly, "proto-oncogenes" play a role in cellular processes related to proliferation and differentiation. When influenced by activating or "gain-of-function" mutations, they become "oncogenes" able to promote or develop cancer [4]. Based on structure and functions, proto-oncogenes classify into tyrosine protein kinase, kinase-related, GTP-binding proteins, growth factor, growth factor receptor, and nuclear proteins [5].

The second group of genes, called TSGs, effectively limit cell growth, differentiation, and apoptosis. This makes sure that cell growth only happens when it needs to and is controlled, which inhibit cancer development [6]. Unlike proto-oncogenes, "loss-of-function" mutations reduce TSG expression in human cancers. Inactive TSGs promote cancer formation and progression by speeding cell proliferation and suppressing apoptosis.

The Inhibitor of Growth (ING) genes are members of TSG family which initially were identified in human cells. Homologs to *ING* genes have been identified in different eukaryotic species from plants to rats such as *Yng* isoforms in *Saccharomyces cerevisiae* sequences [7], while their sequences are known to be well conserved during evolution proposing their substantial functions [8]. They all have been categorized as type-II or gatekeeper TSGs [9]. Based on the “two-hit hypothesis” by Alfred Knudson, two alleles of a TSG are required to be inactivated to allow a cell for tumorigenic excess proliferation [10]. TSGs are classified into five types according to the function of encoded proteins: 1- regulating cell cycle progression, 2- repressing cell proliferation, 3-checkpoint proteins responding to DNA damages, 4- promoting apoptosis, and 5- playing role in DNA mismatch repair [11]. The second class, which a majority of TSGs belongs to, are translated into receptor proteins or signal transducers responding to hormones and stimuli which suppress the cell growth [11]. The best-known example for these is the cytokine TGF-β which acts as a TSG via suppressing the cell proliferation and promoting apoptosis [12]. Other examples are well studied in human malignancies such as APC, BRCA1, p53, and Rb [6].

The first member of the *ING* gene family was reported for the first time by Garkavtsev et al. in 1996 [13]. They employed subtractive hybridization based on a polymerase chain reaction (PCR) technique and then screening the tumor suppressor genes, cloned a newly discovered gene they termed *ING1* with a 33 kDa-encoded protein. *ING1* overexpression caused suppression of Hs578T human breast cancer cell lines, while its inhibition enhanced malignant phenotype of cells and so *ING1* was introduced as a tumor suppressor gene.

Several members of the ING family have been identified with regulatory roles in cell migration, angiogenesis, inflammatory responses, and spermatogenesis. However, these models need to be studied more and verified by experiments. This study discusses what is recently known about ING gene functions and highlight the potential impact of INGs on the development of human cancer.

**Fig. 1:**
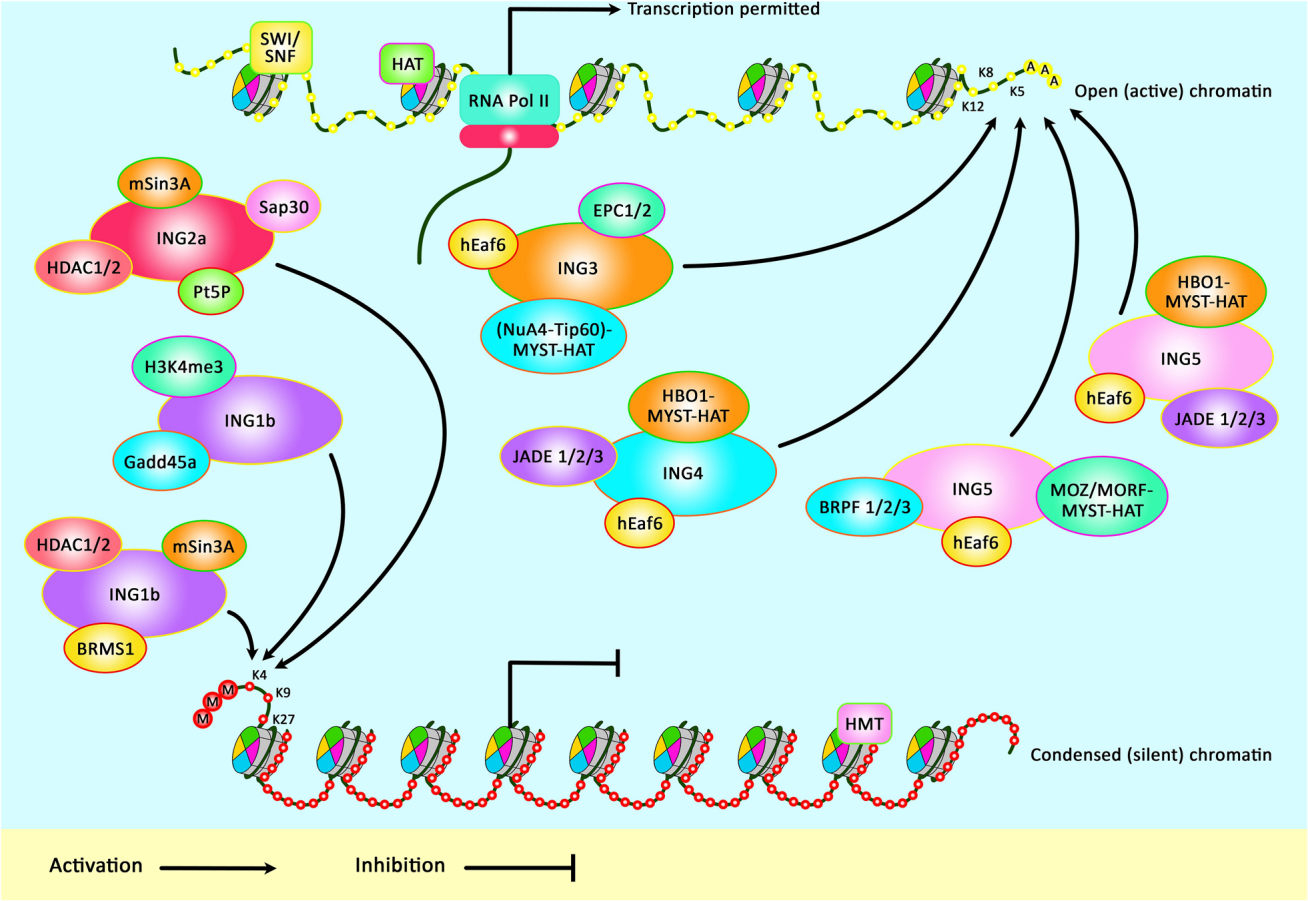
A schematic diagram of the ING protein family members involved in chromatin remodeling. Chromatin compaction is modulated via posttranslational modifications including histone methylation (silent chromatin) as well as histone acetylation (active chromatin). By binding to the histone mark H3K4me3, ING1b recruits the HDAC mSin3A/HDAC1/2 complex or Gadd45a, for local histone deacetylation. ING2a, in complex with HDAC1/2, SAP30 and mSin3A can bind either H3K4me3 or H3K9me3. ING3 can acetylate the N-terminal tails of histones H4 and H2A. Moreover, ING4 and ING5 are in complex with MYST-HB01-JADE-hEAF6-HAT. ING5 is also a component of another HAT complex (MOZ/MORF-MYST-HAT) [14, 15]

Figure 1 illustrates an overview of ING proteins participate in the regulation of chromatin through epigenetic marks.

### Structure and functions of ING proteins

**Fig. 2:**
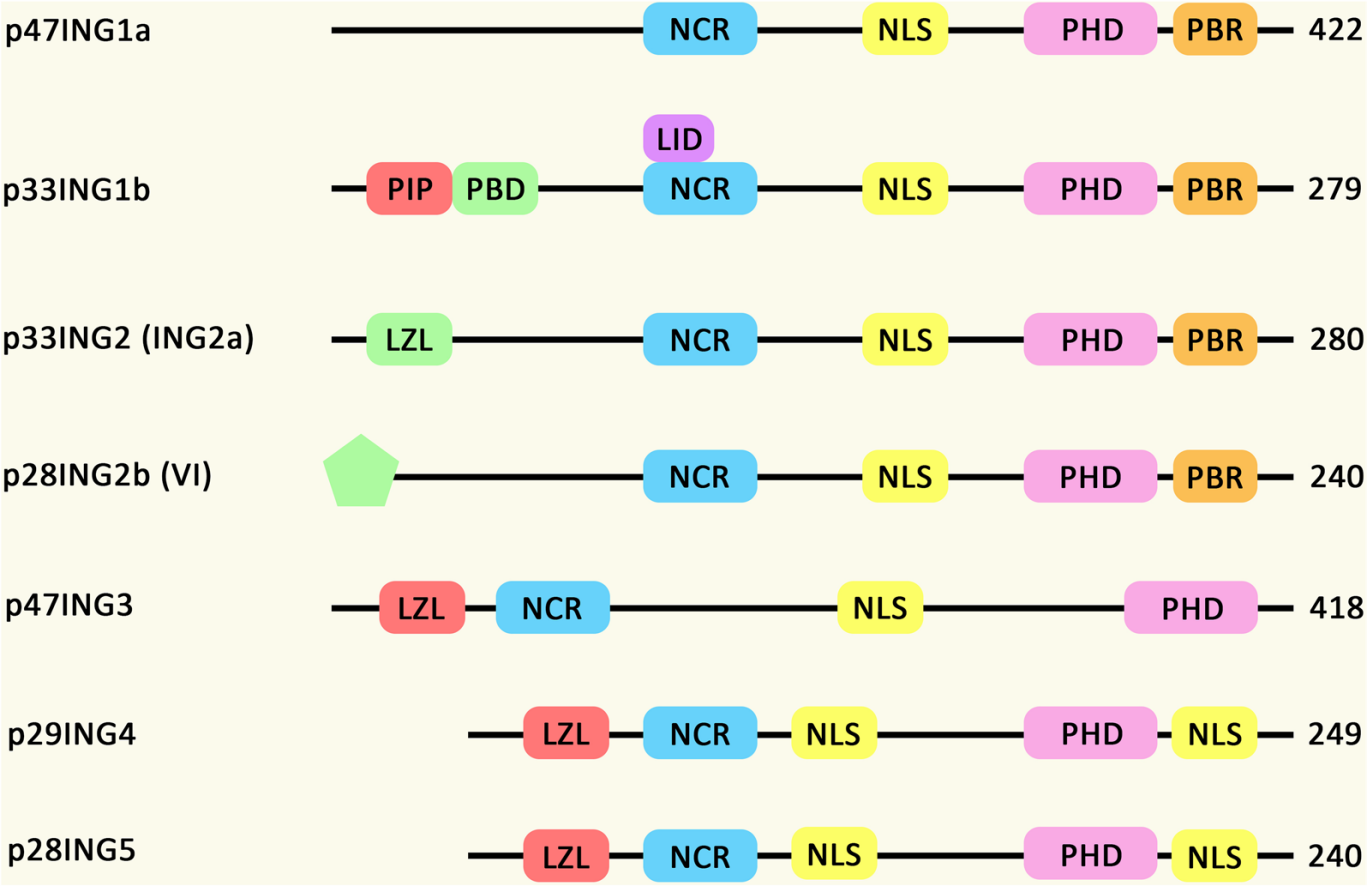
Structural features of ING proteins in Homo sapiens. All ING proteins include three conserved regions, a PHD, NLS, and NCR from C-terminal region to N-terminal region. Moreover, p33ING1b has a PIP, PBD, and LID domains

The ING proteins multidomain proteins, which are mainly conserved among all members and required for their regulatory functions on the cellular growth (Fig. 2) [16]. Through these domains, the ING proteins emerge as binding structures connecting the enzymatic activities to chromatin [17]. Through these interactions, INGs are able to affect pro-proliferative or growth-inhibitory behaviors via epigenetic regulations. Several homologs to human ING proteins have been reported in animals and plants and they all possess N-terminal domains with unique amino acid sequences [16] in addition to a plant homeodomain (PHD) at the C-terminus with conserved sequence between eukaryotic cells [18].

The main domain found in ING proteins is the PHD domain encoding a zinc finger, which is already identified with essential role for the function of many epigenetic regulatory proteins known as chromatin remodeling factors playing role in gene expression [19]. PHD domains act in controlling gene expression via gathering several epigenetic regulatory proteins and transcription factors to the target sequences. Through the PHD, they particularly bind to the histone H3 with 3 methyl groups added at lysine 4 (H3K4me3). H3K4me3 is a mark for active transcription located preferentially near the gene promoters and start sites of loci being actively transcribed [20, 21] and so, ING proteins can impact the expression of several target genes required for activation or inhibition of cellular growth [22]. This is partially conducted through recruiting the “Gadd 45a” factor, which is activated in response to DNA damage and consequently changing the acetylation/methylation of histone residues [23]. In addition to regulation of gene transcription, ING proteins can exert their cell growth-regulatory functions through binding to and altering the activity of TSG p53 and nuclear factor-kappa B (NF-κB) proteins [24].

Sequence conservation of PHD zinc finger domains have helped identification of various *ING* genes among different organisms via phylogenic analyses [16]. On the other side, N-terminal domains of the ING proteins bind to histone modification enzymes such as deacetylase and acetyltransferase (HDAC, and HAT, respectively) proteins, which their balance in adding/removing acetyl groups on the histones play role in epigenetic regulation [25]. Accordingly, ING proteins were initially identified as components of HDAC and HAT complexes [26]. Today, first two ING proteins (ING1, and 2) are considered the subunits of HDAC complex and the latter ING3, 4, and 5 of HAT enzyme. The N-terminal domain is also responsible for a shared potential of all ING proteins in binding to lamin A located in the nuclear envelope [26]. Adjacent to protein-binding domains, ING proteins also have a nuclear localization signal (NLS) or more, which is responsible for their transport to and localization at the nucleus [16]. Additionally, nucleolar translocation sequences (NTS) help translocation of ING proteins stimulated by DNA damage and mutated NTS signals cause decreased apoptotic levels [27].

In the early years of this century, four other members of the ING family (ING2-5) were found using homology-finding methods on their C-terminal domains [28–30]. Their sequences are all very similar to the first member's, which shows that they are all related. Among them, ING1 and ING2 recruit mSin3A/HDAC1/2, and mSin3A/HDAC1, respectively while others demonstrate interaction with various HATs [14]. There are more than 15 different splicing isoforms encoded by the five ING genes. They are all known to have significance in a variety of biological processes, including cell proliferation, apoptosis and senescence, carcinogenesis, DNA repair, and spermatogenesis [31–34]. Accordingly, loss of these genes has been associated with a number of human diseases such as cancers, inflammation, and aging. Furthermore, they have been identified as having dysregulation or inactivating mutations in a variety of human cancers [24].

### ING1

*ING1* was the founding member of the family initially identified in breast cancer cells as a potential TSG and further studied compared to other genes in the past two decades. It is located on the long arm of chromosome 13 near the telomere at 13q34 [35], and its sequence is more similar to ING2 than to other isoforms. This suggests that ING2 and other isoforms came from the same ancestor [16]. Their functions are also more similar [32]. There are 4 variants that are known to be encoded by ING1, and the majority of their expression is found in the p33ING1b and p47ING1a variants [33].

Structurally, *ING1* possesses 4 exons including 1a, 1b (the most common isoform), 1c, and 2 which eventually 4 mRNA variants are developed in transcription via alternative splicing and produced from various promoter regions [32]. These isoforms, which were formerly known as p24^ING1^, p47 ^ING1^, p32 ^ING1^, and p27 ^ING1^ [36], differ in their N-terminal sequences [14], and found to be widely expressed for ING1a and ING1b [37] particularly in high levels of expression in testes for ING1a, and 3 others mainly expressed in thymus and other internal organs [38]. In addition to common ING domains including the PHD, NTS, and NLS, ING1 also possesses a motif mediating the damaged DNA to bind to proliferating cell nuclear antigen (PCNA) [37].

*ING1* was first identified as having tumor suppressor acivity by Garkavtsev et al. [13] who also introduced the founding member of *ING* genes family being expressed in mammary epithelial cells acting as anti-proliferative agents, while not expressed in breast cancer cell lines. The authors demonstrated that p32 ^ING1^ suppresses cell proliferation, and conversely its inhibition using antisense RNA showed opposite effect on cell growth of breast cancer cells [13]. In addition to acting as a TSG with growth-suppressive properties, ING1 can also trigger apoptosis and participate in cellular senescence [39, 40]. In normal neurons, ING1 plays a role in regulating activity-dependent gene expression [41]. Furthermore, upregulation of ING1 increases Bax activity and modifies mitochondrial membrane potential through a process that requires p53 in the cell [42].

In addition to its involvement in controlling cell development in non-cancerous cells, ING1 regulates a number of pathways in malignant cells. Recent studies showed that ING1 gene expression has been found to be lost in a variety of human malignancies, either alone or in combination with protein expression, showing that it is downregulated in cancer tissues compared to healthy tissues. Some studies even reported that its expression in breast cancer cell lines has decreased by 100% [43]. The absence of ING1 protein may result from a decrease in either its expression or the stability of its mRNA [9]. However, ING1 loss of expression without its alterations in human malignancies has not been observed [14, 44].

ING1's role as a tumor suppressor gene (TSG) has been demonstrated in a variety of human cancers including lung cancer [45], colorectal cancer [46], prostate cancer [38], and astrocytoma [47]. Further study has found that ING1a regulates cell senescence while ING1b triggers apoptosis [48, 49]. In PCa cells, ING1b silencing has also increased cellular senescence and induced expression of the effective cell cycle inhibitor p16INK4a [50].Conversely, upregulation of ING1 has been shown to inhibit cell growth and metastasis in breast cancer in vitro and in vivo [51].

Interestingly, ING1 expression is regulated by several mechanisms including epigenetic regulation such as methylation [38, 52], effect of microRNAs [53], and post-translational modifications [54]. Besides that, ING1 itself plays a role in tumor suppression by affecting several cancer hallmarks, such as sustained cell proliferation, growth and metastasis, evading cell apoptosis, and angiogenesis, through epigenetic regulatory mechanisms and interactions with other TSGs such as p53 [14]. For instance, based on experiments with overexpression, ING1 can regulate wild-type p53 but not mutant p53 in MDM2-independent. Knocking down endogenous ING1 lowered p53 levels in a transcription-independent manner [55].

### ING2

Similar to *ING1*, *ING2* is also located near the telomeric region but on chromosome 4q35.1. As stated for ING1, these two have a high level of homology in their amino acid composition [28], which suggests that they share a common ancestor [16] and have a close evolutionary and functional relationship [26].

ING2 protein has two isoforms of ING2a (mainly referred as ING2) and ING2b resulted from alternative splicing between 2 exons of 1a, and 1b among 3 exons of its encoding gene (1a, 1b, and 2). ING2 was initially identified in human fetal brain termed ING1 homologous (INGL) [56]. It has another domain in addition to other common ING domains which is reported for other isoforms except ING1. This domain is leucine zipper-like (LZL) which possesses 4–5 conserved leucine/ isoleucine amino acids which form a hydrophobic patch at the N-terminal end of protein [18]. LZL is specifically required for proper functions of ING2 including apoptosis, nucleotide excision repair, and chromatic remodeling in exposure to UV light [57]. It is thought that the p53 tumor suppressor is responsible for these effects. In response to DNA damage, ING2 acts as a nuclear phosphoinositide receptor [58]. This suggests that ING2 has a direct role in DNA damage response. Through its PHD domain, ING2 interacts with phosphatidylinositol 5-phosphate, which activates p53 and leads to apoptosis [58]. ING2 is a subunit of mSin3A-HDAC corepressor complexes and it controls gene expression by binding to H3K4me3.

In addition to characteristic growth-inhibitory effects, ING2 is known to be involved in several other processes like spermatogenesis and muscle differentiation [59, 60]. Saito et al. [59] demonstrated that ING2-deficient male mice were infertile and had defect spermatogenesis with significantly decreased sperm number and testicular structural abnormalities. These results were seen concomitant lack of p53, and the authors concluded that ING2 in an axis of chromatin regulation via HDAC1/ING2/H3K4me3 plays role in spermatogenesis. Accordingly, lack of ING2 expression has been reported with loss of function in a variety of cancers such as lung cancer and head and neck squamous cell carcinoma [61, 62]. Same to ING1, ING2 affects cancer development via epigenetic mechanisms.

### ING3

ING3 possesses the most distinctive sequence compared to other ING family members since ING1 and ING2 and also ING4 and ING5 demonstrate high similarity, while ING3 shows the lowest amount of similarity to other [16]. Additionally, unlike others, *ING3* gene is not located at the telomeric region [63]. It is located at chromosome 7q31.3, possesses 12 exons encoding a protein with 418 amino acids [64]. Doyon et al. [63] reported that ING3 is an essential subunit of NuA4-Tip60 MYST-HAT protein complex acting in chromatic regulation. ING3 shows extensive expression in a wide variety of mammalian tissues such as human heart, spleen, and muscles and mice kidney, cardiomyocyte, and skeletal muscles; however, highest levels have been reported in oocytes [64–66]. It is found to regulate cell growth, control cell cycle, modulate p53 transcription, and induce apoptosis [64].

Accordingly, ING3 loss of expression has been reported in a number of human cancers, such as gynecological malignancies [66], where its high expression at the nucleus of cancer cells is reported to be associated with good prognosis in breast cancer patients and downregulation of its mRNA correlates with poor prognosis in head and neck malignancies [67, 68]. Li et al. [69] demonstrated that ING3 overexpression inhibits cell migration and invasion in breast cancer cells regulating cancer behaviors through tumor-associated pathways. Additionally, Melekhova et al. [69] showed that knocking down ING3 in a variant without a PHD domain leads to more epithelial-to-mesenchymal transition (EMT) and cellular senescence in the LNCaP prostate cancer cell line. This means that this TSG decreases the ability of cancer cells to spread.

Almost all in vitro studies show that ING3 is a TSG with a growth-inhibiting function. However, in prostate cancer, Nabbi et al. found that ING3 works as a co-activator of androgen receptor (AR) [70]. Thus, ING3 acts also as a coactivator being distinct from ING1 and ING2. It was reported that each ING1b and ING2a functions as corepressor of AR in prostate cancer cells, cross-talk and compensate their activity if one is downregulated. Interestingly, each is able to induce cellular senescence in cancer cells [71].

Overall, ING1 and 2 are identified as AR-corepressors inhibiting androgen signaling, while ING3 is known to act as AR-coactivator playing role in prostate cancer pathogenesis [72, 73]. In addition to growth-regulatory roles, ING3 is found to play role in embryonic growth and its loss associated with disturbance in neural tube development, ectodermal differentiation, and death in mice embryos [74]. This suggests that it is involved in cell migration and differentiation in brain development.

### ING4

ING4 was initially reported using homology studies and Shiseki et al. characterized it in human placenta [75]. It is located near the telomeric region, on chromosome 12p13.31, and the gene encoding this protein has 1380 nucleotides and 8 exons [37]. ING4 protein contains 249 amino acids forming a 29-KDa protein with several domains like PHD, LZL, and NLS and high homology in sequence compared to the last family member; ING5 [76].

ING4 has been found to be expressed in all tissues of the mammalian body. It is also a member of HB01-JADE-hEAF6 MYST-ING HAT protein complex sensing histone marks and playing role in chromatin remodeling [26]. These effects are exerted through binding to H3K4me3 and HBO1-JADE via its PHD domains [77]. ING4 also binds to p53 via its NLS domain, thus it is suggested to play role in tumor suppression partly through interacting with p53 tumor suppressor [77].

Furthermore, ING4 is known to regulate several other processes like apoptosis, autophagy, angiogenesis, DNA repair, and malignant phenotype of cancer cells [78]. Accordingly, loss of ING4 expression and pathogenic mutations have been reported in various types of human cancer cells, which support its role as a TSG regulating cell growth playing role in avoiding cancer development [79–82]. Low expression of ING4 in the cancer cells is accompanied cycle cell arrest at the G2/M phase demonstrating its role in regulation of cell cycle [26]. Additionally, Yang et al. [83] reported that ING4 acts as an anti-inflammatory factor through affecting NF-κB pathway via having interaction with Sirtuin1 (SIRT1) in lipopolysaccharide-induced sepsis. Consistent with this finding, Qian et al. [84] demonstrated that ING4 suppresses hepatocellular carcinoma (HCC) via repressing transcriptional activity of NF-κB, upregulation of miR-155 targeting FOXO3a in MHCC97H human HCC cells.

### ING5

ING5 is located at chromosome 2q37.3 encoding a protein composed of 240 residues forming a 28-kDa protein [85]. ING5 is the last member of ING protein family as TSGs with inhibitory roles in tumor development and progression. In addition to growth-inhibitory effects, ING5 is shown to regulate differentiation of stem cells such as epidermal stem cells and cancer stem cells [86, 87]. ING5 also was initially identified through homology searching approaches [26]. In addition to several other domains like LZL, and NLS, ING5 possesses a PHD domain associates with HAT complexes and demonstrates controversial effects as a TSG or oncogene [14, 88].

In several cancer tissues acute myeloid leukemia (AML) and head and neck cancer, ING5 is reported to be downregulated suggesting it with anti-tumor effects. However, in a few studies changes of ING5 at mRNA and protein levels are reported inconsistently or being upregulated in cancer tissues [89–91]. Importantly, ING5 is known to affect several cell processes like cell proliferation, autophagy, and apoptosis. It also has a negative effect on the malignant phenotype of cancer cells [92], which suggests that it controls a lot of different growth-controlling pathways within the cells. In an interesting study, ING5 changes the way miRNAs work. Cui et al. [90] showed that miR-24 acts as an upstream oncogenic miRNA that suppresses ING5 expression and has the opposite effect on cell proliferation, invasion, and apoptosis in breast cancer cell lines compared to ING5. ING5 is particularly associated with p53 and through activating this tumor suppressor can fight against malignancies as a TSG [93]. Furthermore, ING5 is known to regulate signaling pathways like AKT and inflammatory pathways playing role in development and progression of cancers [94, 95].

**Fig. 3:**
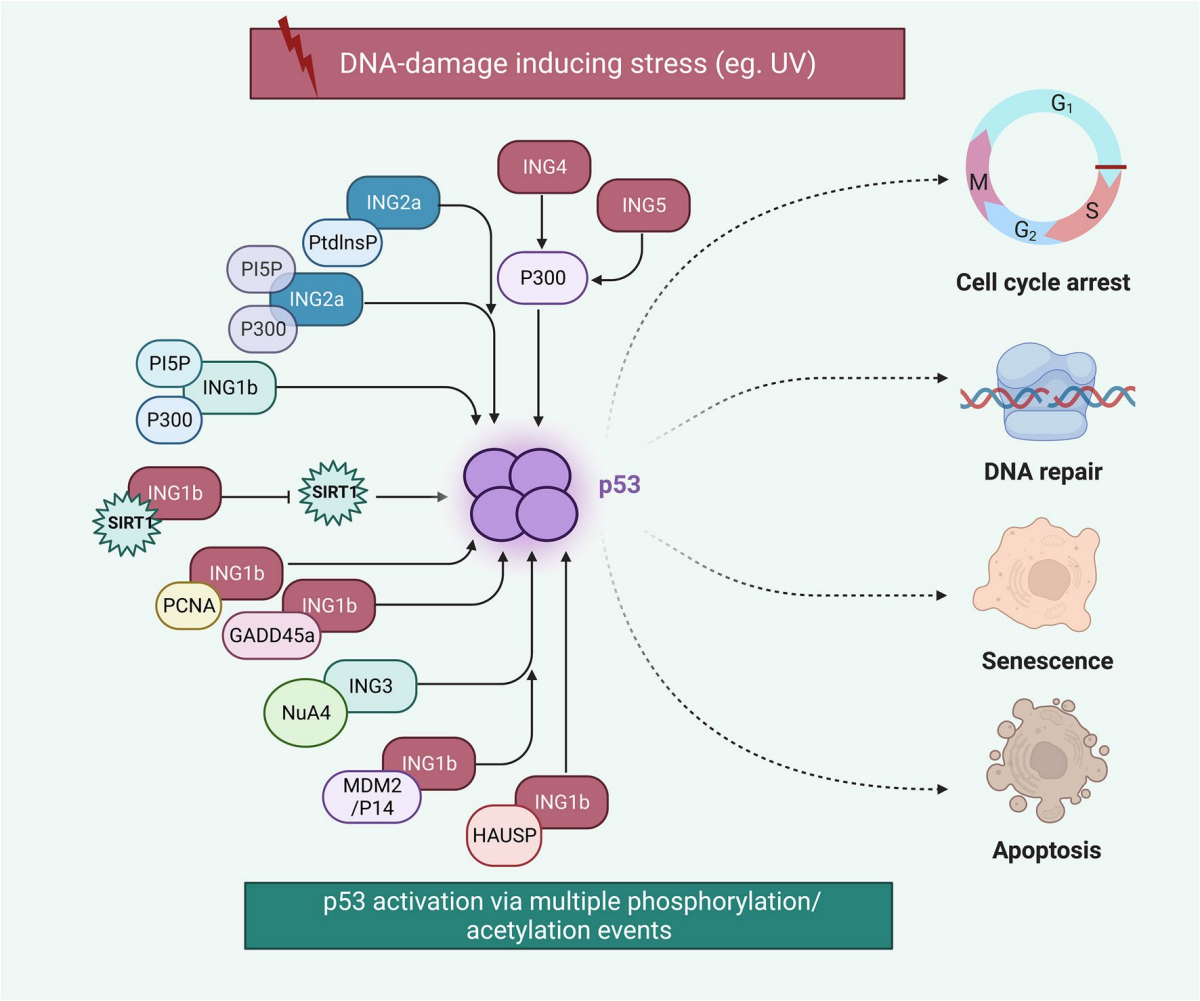
A schematic illustration of the ING proteins activating the p53 cascade. All members of the ING family (INGs 1–5) have been detected to associate with enhancing p53 activity via inducing its acetylation or increasing its stability. ING proteins differentially impinge upon the main tumor suppressive cascades of apoptosis, anti-proliferation, cell cycle arrest as well as senescence [32, 98, 99]

INGs have interactions with p53. For instance, two p53 binding sites have been identified in *ING2a* promoter possesses. Moreover, Nutlin-3-mediated activation of p53 could suppress ING2a transcription [96]. ING2 has also been shown to negatively regulate cell proliferation via inducing acetylation of p53 [97]. Another study has shown induction of apoptosis in ING2 − / − germinal cells and enhancement in their p53 protein levels possibly due to induction of testicular degeneration in the absence of ING2 or presence of a regulatory interaction between ING2 and p53. Further experiments have shown that ING2 deficiency in testis stimulates both p53-dependent and independent apoptotic pathways [59]. Both ING1b and ING2a can regulate acetylation of p53 to control cell cycle transition, cell apoptosis, and senescence [32]. Figure 3 demonstrates the regulation of p53 function by ING family members in inhibition of tumor initiation and development.

## Concluding remarks

ING family including 5 members (ING1-5) have been known as candidate TSGs with growth-inhibitory effects in addition to other restrictive influences on malignant phenotype of cancer cells. They are also known to act in several other cellular processes like spermatogenesis, differentiation of stem cells, and cell senescence. INGs are subunits of HAT/HDAC protein complexes involved in chromatin regulation through which they are believed to exert their regulatory functions. Particularly, they are known to be downregulated in human malignancies, while their overexpression can provide therapeutic potential for cancer treatment. Based on their sequence similarities and protein domains structures it suggests ING family members have overlapping and redundant activities. The knock-out or knockdown of one member might be compensated by another family member. Thus, double knock-out or knock-down experiments may shed further light into their biological roles in health and disease.

In brief, INGs represent a group of proteins with substantial roles in the carcinogenesis. These proteins can be used as potential targets in desing of anticancer modalities. Yet, there is still limited data about application of INGs in therapeutic options. Future in vitro and in vivo studies are needed to elaborate the exact mechanisms of participation of INGs in the carcinogenesis, their interactions with other tumor suppressors or oncogenes and functional consequences of their ablation. High throughput sequencing methods would help in indentification of specific targets of INGs and recognition of the underlying mechanisms of their participation in the carcinogenesis.

## Data Availability

The analyzed data sets generated during the study are available from the corresponding author on reasonable request.

## Abbreviations

ING: Inhibitor of growth
TSGs: Tumor suppressor genes
PCR: Polymerase chain reaction
PHD: Plant homeodomain
H3K4me3: H3 with 3 methyl groups added at lysine 4
NF-κB: Nuclear factor-kappa B
NLS: Nuclear localization signal
NTS: Nucleolar translocation sequences
LZL: Leucine zipper-like
EMT: Epithelial–mesenchymal transition
AR: Androgen receptor
SIRT1: Sirtuin1
HCC: Hepatocellular carcinoma
AML: Acute myeloid leukemia
